# Acupressure therapy and Liu Zi Jue Qigong for pulmonary function and quality of life in patients with severe novel coronavirus pneumonia (COVID-19): a study protocol for a randomized controlled trial

**DOI:** 10.1186/s13063-020-04693-5

**Published:** 2020-08-27

**Authors:** Shuaipan Zhang, Qingguang Zhu, Chao Zhan, Wei Cheng, Xiao Mingfang, Min Fang, Lei Fang

**Affiliations:** 1 Yue yang Hospital of Integrated Traditional Chinese and Western Medicine, Shanghai University of Traditional Chinese Medicine, Shanghai, 200437 China; 2 Huangshi Hospital of Traditional Chinese Medicine, Huangshi, Hubei 435000 China; 3 School of Acupuncture-Moxibustion and Tuina, Shanghai University of Traditional Chinese Medicine, Shanghai, 201203 China; 4 School of Rehabilitation Science, Shanghai University of Traditional Chinese Medicine, Shanghai, 201203 China

**Keywords:** COVID-19, Acupressure therapy, Liu Zi Jue Qigong, Traditional Chinese medicine rehabilitation, Randomized controlled trial, Study protocol

## Abstract

**Background:**

In December 2019, pneumonia associated with the 2019 novel coronavirus (COVID-19) emerged in Wuhan, China. The number of cases has increased rapidly. Patients with severe disease have a poor prognosis, and there are no effective therapies for COVID-19. Only rapid advice guidelines for symptomatic supportive care have been used. A traditional Chinese medicine rehabilitation (TCMR) program consisting of acupressure therapy and Liu Zi Jue Qigong can be used as a complementary therapy for COVID-19. Hence, we designed a randomized trial to evaluate the efficacy and advantages of TCMR for treating patients with severe COVID-19.

**Methods/design:**

This is a parallel-design, two-arm, analyst assessor-blinded, randomized controlled trial. A total of 128 patients with COVID-19 aged from 20 to 80 years will be recruited and assigned randomly into a guideline therapy group and a guideline therapy plus TCMR group at a 1:1 ratio. Patients in both groups will receive guideline therapy. The patients in the intervention group will perform acupressure therapy and Liu Zi Jue Qigong exercises in addition to conventional treatments twice a day and will be persistent from admission to discharge. The primary outcome will be measured with the Modified Medical Research Council Dyspnea Scale, and the secondary outcomes will include the Activities of Daily Living Barthel Index Scale, Patient Health Questionnaire-9 Scale, and the Respiratory Symptoms Scale. The assessments of the clinical scales will be performed at three points (before treatment, the 7th day during hospitalization, and the discharge day). Adverse events will be noted and recorded for the safety evaluation.

**Discussion:**

This trial will provide high-quality evidence of the value of TCMR, which consists of acupressure therapy and Liu Zi Jue Qigong exercises, for treating patients with severe COVID-19.

**Trial registration:**

Chinese Clinical Trial Registry ChiCTR2000029994. Registered on 18 February 2020

## Background

Since December 2019, some patients with pneumonitis infected by a new type of coronavirus called COVID-19 were successively found in Wuhan, which raised intense attention worldwide [1]. COVID-19 is a novel type of coronavirus belonging to the family beta-coronavirus, and its gene sequence is significantly different from previous viruses [2]. COVID-19 is mainly transmitted through respiratory droplets and contact and has high infectiousness. Though the authorities gave the highest priority to prompt prevention and treatment of COVID-19, cases of human-to-human transmission are gradually expanding domestically and abroad. As of 27 May 2020, more than 5,406,282 people have been infected, and more than 343,562 deaths have occurred globally [3]. Patients often develop dyspnea or hypoxemia 1 week after the onset of symptoms, and in severe cases, they progress rapidly to acute respiratory distress syndrome, septic shock, etc. [4]. An early study proved that due to a shortage of medical resources, the mortality rate of patients with severe pneumonia is as high as 61.5% [5]. Eventually, the National Diagnostic and Treatment Protocol for Novel Coronavirus Pneumonia (the 7th trial version) was developed to reduce mortality and reinfection [6]. However, more options should be explored to cope with this event [6, 7]. Acupressure is a low-risk physical therapy that is different from acupuncture and is a part of traditional Chinese medicine (TCM). Previous research has shown that acupressure can improve patients’ symptoms of dyspnea in lung diseases and their quality of life [8, 9]. Acupressure is a noninvasive treatment accepted by patients; this treatment is characterized by pressing on acupoints with the hands to achieve clinical efficacy. In addition, Liu Zi Jue Qigong exercises have also been widely used in lung rehabilitation training; these exercises not only relieve symptoms of breathlessness but also have benefits on mental illness [10–12]. Liu Zi Jue Qigong is applicable for the patients with COVID-19 who widely suffer panic and anxiety due to the mortality and infectivity of the disease [13]. These exercises adopt a combination of abdominal breathing and lip breathing to produce six different sounds (xu, he, hu, si, chui, and xi) accompanied by low-intensity body movements [12]. This breathing pattern can change the rapid shallow breathing pattern of lung dysfunction, extend the opening time of the trachea, and maintain the airway pressure of the patient within a physiological range, thereby improving gas exchange [14, 15]. Regarding COVID-19 patients, dyspnea is the most obvious clinical symptom. Furthermore, sudden illness and fear of disease are psychological disorders that each patient will have. Previous studies have shown that acupressure and Liu Zi Jue exercises can improve the respiratory symptoms of patients with lung disease as well as improve their quality of life and mental health. Therefore, effectively combining the two interventions into a rehabilitation method can potentially be used in the clinical rehabilitation of COVID-19 patients. Now, we will combine the acupressure therapy with Qigong exercises as a clinical traditional Chinese medicine rehabilitation (TCMR) method for patients with severe pneumonia. Therefore, we hypothesize that official conventional therapy plus TCMR will have better clinical efficacy than single conventional therapy for clinical symptoms, mental health, and quality of life.

## Methods/design

### Study design

**Fig. 1:**
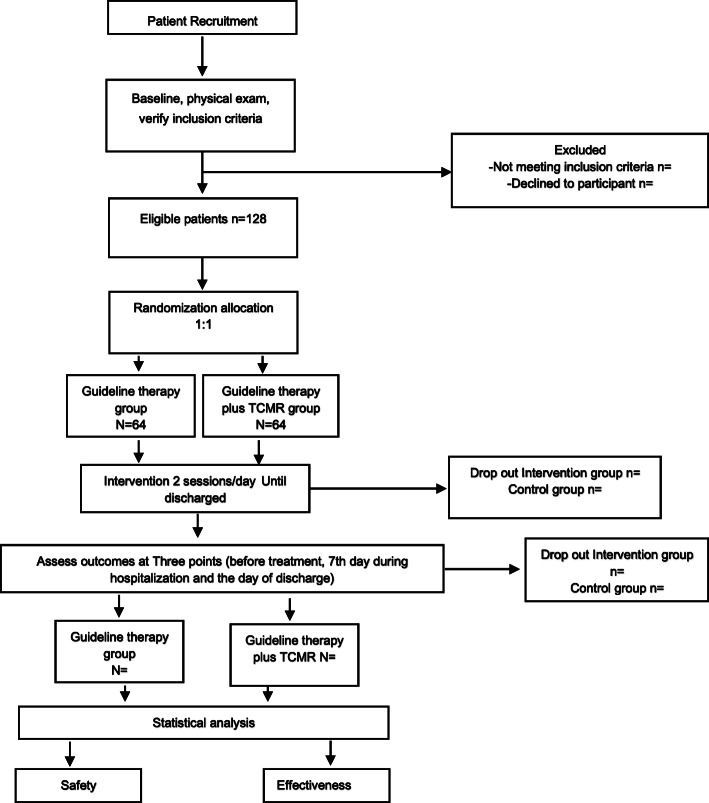
Flow chart of the trial

**Fig. 2:**
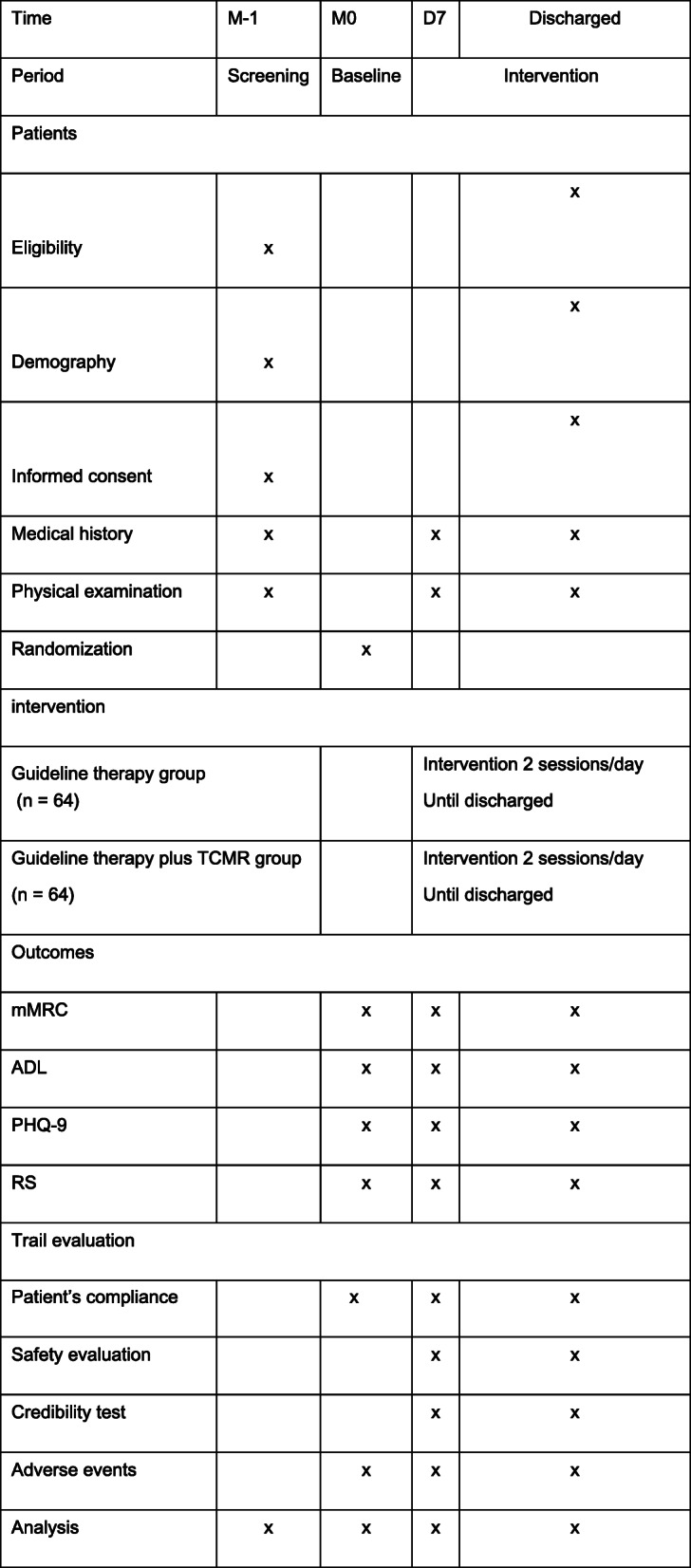
Study schedule showing the time points for enrollment and assessment. ADL, activities of daily living; CRF, case report form; CT, computed tomographic; mMRC, modified Medical Research Council; PHQ-9, Patient Health Questionnaire-9; RS, respiratory symptoms; TCMR, traditional Chinese medicine rehabilitation

This will be a single-center, parallel-arms, superiority randomized controlled trial (RCT). The protocol was registered with the China Clinical Trial Registry (item number: ChiCTR2000029994), and the trial protocol was approved by the Ethics Committee of Huangshi Hospital of Traditional Chinese Medicine (item number: HSZYYJ-2020-003-01). A total of 128 patients will be recruited from the Huangshi Hospital of Traditional Chinese Medicine in Hubei province. Written informed consent will be provided by all patients at the time of recruitment. Patients with severe pneumonia symptoms will be recruited and will have equal chances of being allocated randomly to guideline therapy or guideline therapy plus TCMR. Due to the limitations of the intervention methods, only the outcome assessors and statisticians will be blinded. The assessor will perform evaluations and analysis of the outcomes at three points (before treatment, the 7th day during hospitalization, and the discharge day). Data management and statistics will be conducted at the Rehabilitation Medical College of Shanghai University of Traditional Chinese Medicine (SUTCM). The flow chart in Fig. 1 illustrates the study design, and Fig. 2 shows the study schedule. Additional file 1 shows the Standard Protocol Items: Recommendations for Interventional Trials (SPIRIT) checklist.

### Participant recruitment

Patients with severe COVID-19 will be recruited; they will meet the National Diagnostic and Treatment Protocol (the 7th trial version). The participants will be from the Department of Infectious Diseases at Huangshi Hospital of Traditional Chinese Medicine. Recruitment advertisements will be placed on the inpatient, network, WeChat, and Weibo official platforms to enroll potential participants. The informed consent process will be conducted by the principal investigator (LF) or study coordinator (WC) who will screen participants to confirm that potential participants meet the eligibility criteria listed below if the participants agree to sign the informed consent form.

### Inclusion criteria

Patients will only be recruited if they meet each of the following conditions: (1) meet the critical diagnosis criteria for severe COVID-19 [6], (2) are aged between 20 and 80 years and are male or female, (3) have a stable condition and are conscious and cooperative in the examination, (4) volunteer to join the trial and sign the informed consent form, and (5) promise not to perform other exercise programs.

### Exclusion criteria

Those who meet one of the following conditions will be removed: (1) patients with complications of other serious underlying diseases, such as chronic obstructive pulmonary disease, obstructive pulmonary disease, coronary heart disease, and hypertension; (2) patients with serious mental illness; (3) patients with cognitive dysfunction who are unable to understand the trial process and rehabilitation content; (4) patients with severe bone and joint diseases (such as spinal arthritis, severe osteoporosis, and periarthritis) affecting limb function and movement; (4) patients with respiratory failure who require mechanical ventilation or patients with shock or combined organ failure requiring intensive care unit (ICU) monitoring and treatment; (5) pregnant or lactating women; and (6) patients who participate in other forms of exercise during the trial.

### Dropout and suspension criteria

During the intervention period, patients will have the right to withdraw for whatever reason and at whatever time under the protection of the Declaration of Helsinki [16]. In addition, if the hospitalization time is less than 7 days, the subject will be excluded as the outcome evaluation cannot be completed.

### Interventions

For the emergency event of COVID-19, standard therapy has been developed in strict accordance with the National Diagnostic and Treatment Protocol (the 7th trial version). For patients, standard therapy is necessary to prevent death and other adverse consequences. On the basis of the guideline therapy, the test group will require a standardized TCMR program consisting of acupressure therapy and Liu Zi Jue Qigong exercises. The program will be performed twice a day at 10 am and 4 pm. Each time, acupressure will be performed first, and then Liu Zi Jue Qigong exercises will continue. Each treatment will last 20 min; thus, a total of 40 min will be needed. The TCMR program will be ongoing during the patient’s stay until the patient is discharged. There will be consensus in advance regarding the acupuncture points, pressure levels, and duration. Liu Zi Jue Qigong exercises will be demonstrated live by the therapists on the first day until the patient is able to perform the exercises skillfully. Patients will be provided with paper and video versions of the exercises to facilitate subsequent practice. Therapists performing rehabilitation therapy will be required to have 10 years of clinical experience in acupressure and Qigong therapy and have undergone rigorous clinical trial training before conducting trials until passing an exam to perform the intervention. Furthermore, both groups of the participants will be allowed to participate in normal exercise under the intervention conditions.

### Guideline therapy

The health authorities have drafted a conventional treatment plan for patients with severe conditions; this is described below. (1) Patients should ensure adequate rest time, and medical staff need to closely monitor vital signs, oxygen saturation, etc. (2) Additionally, it is necessary to monitor routine blood and urine tests, C-reactive protein, biochemical indicators, blood coagulation function, arterial blood gas analysis, chest imaging, etc. according to the changes in the condition. (3) Timely effective oxygen therapy measures should be taken; in particular, patients with severe disease should receive a nasal cannula or mask to inhale oxygen, and whether respiratory distress and/or hypoxemia is relieved should be promptly evaluated. (4) Doctors should use antiviral drugs combined with antibiotics if necessary. (5) To maintain the stability of the internal environment, they should strengthen supportive treatment to ensure sufficient heat and pay attention to water and electrolyte balance. (6) In addition, early Chinese medicine can be used to relieve syndromes.

### Guideline therapy plus TCMR

Conventional treatment as the authorities have required will be the same as that of the control group. In addition, acupressure therapy and Liu Zi Jue Qigong exercises will be carried out in the TCMR program.

#### Acupressure therapy

**Fig. 3:**
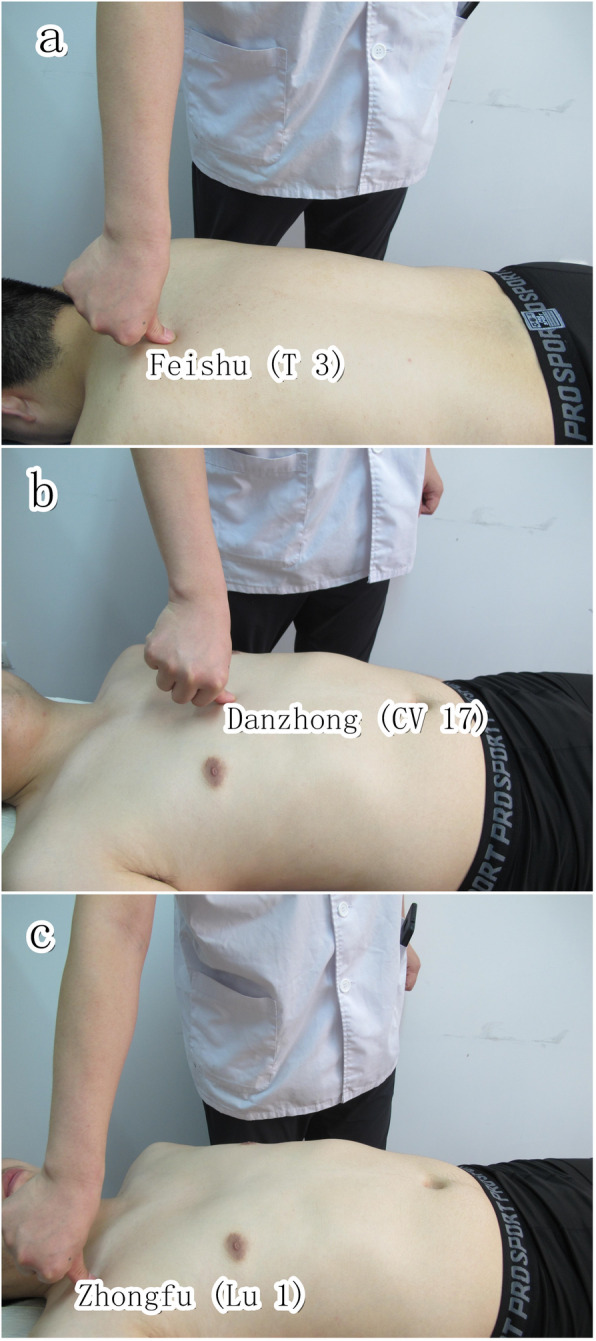
Acupressure operation

The specific acupressure process will be as follows. According to channel-collateral theory, severe cases display a lung qi deficiency pattern characterized by a weak cough, dyspnea, low voice, and clear watery sputum with other symptoms of qi deficiency. Therefore, acupoints related to the lung viscera including Feishu (T 3), Danzhong (CV 17), and Zhongfu (Lu 1) will be selected [17]. Pressing these points can improve lung symptoms [3]. First, the patient will take a sitting or lying position with the therapist on the patient’s right side. Pressing and kneading constitute the manipulation of acupressure. The first step is called the continuous shiatsu method. The therapist uses the thumb pad to press on the selected acupuncture points with moderate force, which is approximately 10 N as tested by a mechanical instrument [18]. The direction of the pressure should be perpendicular to the skin surface, and the pressure is held for 3–7 s and then relaxed without leaving the skin surface. The second step is kneading the acupoint with the thumb. The thumb acts on the skin surface of the acupoint with a slight force pressure (5 N) and then performs a small circular sliding movement with the acupoint at the center. This procedure is performed 50 times on each acupoint with the pressing method and kneading method. The acupressure operation is shown in Fig. 3.

#### Liu Zi Jue Qigong exercises

**Fig. 4:**
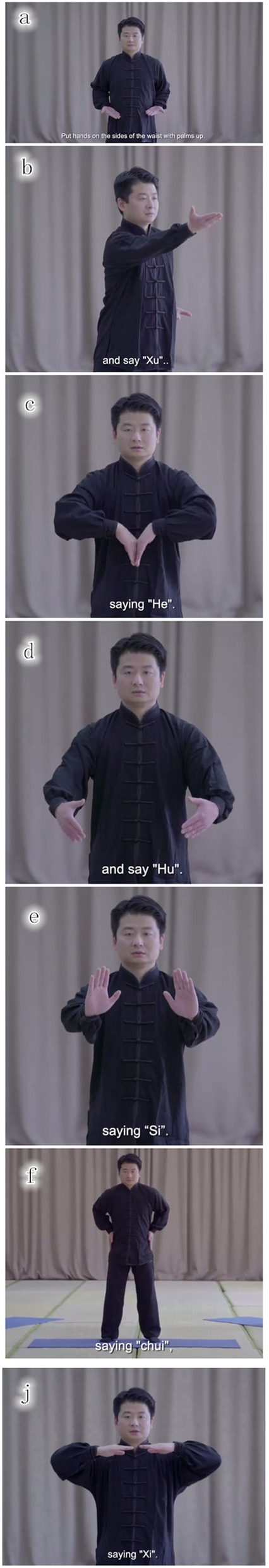
The basic seven movements of the exercise

Liu Zi Jue Qigong exercises are performed as follows: (1) Preparation phase—the patient is standing with the body in a relaxed state, and abdominal breathing or nasal suction is used to relax the abdominal muscles. The diaphragm is contracted together without breathing for 3 s. (2) Practice phase—whistling with the lips is performed to make the “xu, he, hu, si, chui, and xi” sounds lasting for 5 s each. After each of the 6 pronunciations, the patient returns to the ready posture and then performs the next exercise. Each voice exercise is repeated 12 times. The basic seven movements of the exercise are shown in Fig. 4.

### Adherence

Acupressure therapy will be conducted by the physiotherapists every day, and they will complete a recording daily. The nurse will inform the patient to perform Liu Zi Jue exercises every day at 9 am and 4 pm. Each ward has remote monitoring so that remote monitoring of the training of the subject can be achieved, and the participants will be required to sign in a diary after each exercise. Eventually, participants who can complete the training will be rewarded with monetary rewards.

### Plan for retaining participants

An adequate retention plan is a valuable component of the research design process that can enhance participants’ ties with the researchers and the study [19]. First, we will establish a local retention working group including the research coordinator and nurses. The coordinator and nurse will be fully trained before the trial starts; by focusing on daily communication strategies, they can improve retention after receiving additional attention [20]. Furthermore, it is useful to provide sufficient funds within the study budget to compensate the nurses and participants for retention. Subjects will be monitored from a distance while completing their daily physical therapy with a diary recording.

### Outcome measurements

All outcomes will be managed by researchers masked to the group assignment; the outcomes will include the Modified Medical Research Council Dyspnea Scale, Activities of Daily Living Barthel Index Scale, Patient Health Questionnaire-9 Scale [21], and Respiratory Symptoms Scale. Evaluation of the outcomes will occur at three points (before treatment, the 7th day during hospitalization, and the discharge day).

### Primary outcome measurements

#### Modified Medical Research Council (mMRC) Dyspnea Scale

We will use the mMRC as the primary outcome indicator to evaluate the dyspnea symptoms and physical health; this scale is a good indicator of the functional capacity of patients’ lungs [22]. Evaluation of the outcomes will occur at three points (before treatment, the 7th day during hospitalization, and the discharge day). The mMRC [22, 23] grades are as follows: mMRC grade 0, dyspnea occurs only during strenuous exercise; mMRC grade 1—the patient has shortness of breath when walking on flat ground or walking on a small slope; mMRC grade 2—due to shortness of breath, when walking on flat ground, the patient is slower than another person of the same age or needs to stop to rest; mMRC grade 3—when walking on flat ground for approximately 100 m or after a few minutes, the patient needs to stop to pant; and mMRC grade 4—due to severe breathing difficulties, the patient cannot leave the house or has breathing difficulties when dressing or undressing. We will assess the mMRC with a repeated longitudinal analysis.

### Secondary outcome measurements

#### Activities of Daily Living Barthel Index (ADL-BI) Scale

The Barthel Index (BI) is one of the most commonly used methods to assess a person’s basic self-care ability and the intensity of care required; this measure is better suited to acute settings [24–26]. If the total score is greater than 40, the patient is considered more capable. The BI mainly includes 10 daily life behaviors, such as eating, bathing, dressing, and controlling bowel movements. Each item can be scored 10 points if it can be completed independently. Five points are needed for partial help. A total score of 100 indicates that the patient can perform daily activities and does not need to depend on others. A score of > 60 is considered good, indicating that the patient has mild dysfunction and is able to take care of his- or herself in basic daily life. A score of 60–41 indicates moderate dysfunction, and it is obvious that in daily life, the patient depends on others. A score < 20 indicates that the patient is completely disabled, and his or her daily life is completely dependent on others. A repeated longitudinal analysis will also be used to evaluate the ADL-BI.

#### Patient Health Questionnaire-9 (PHQ-9) Scale

The PHQ-9 is used to detect and measure depression and severity in medical populations in clinical settings; this scale has been implemented in a variety of settings using medical populations. It is a questionnaire evaluating the frequency of 9 negative items that happened to patients in the last 2 weeks. If the answer is not at all, the score is 0 points; if once every few days, the score is 1 point; if once more than half a day, the score is 2 points; and if every day, the score is 3 points. The PHQ-9 score is divided into the following categories of increasing severity: 0–4, 5–9, 10–14, 15–19, and 20 or greater. The PHQ-9 results will be assessed using a repeated longitudinal analysis.

#### Respiratory Symptoms (RS) Scale

Respiratory symptoms include fatigue, cough, expectoration, chest tightness, dyspnea, sore throat, nasal congestion, runny nose, and some other nonspecific symptoms. The severity of these symptoms is recorded sequentially as 0–10, with 0 being no and 10 being the most severe. These are self-evaluation scales that will be evaluated with a repeated longitudinal analysis.

### Sample size calculation

The PASS software (PASS 11, NCSS, LLC, Kaysville, UT, USA) will be used to estimate the sample size utilizing two independent sample means (*α* = 0.05, *β* = 0.10). We will use the mMRC as the primary efficacy outcome. Based on previous clinical studies on the effect of respiratory symptoms after acupressure therapy plus Liu Zi Jue Qigong interventions, the mMRC Scale score in the control group is 0.52 with a standard deviation of 0.11 [27], and the average mMRC Scale score in the treatment group is 0.95 with a standard deviation of 0.92. In this study, the target sample size will be 64 participants in each group, anticipating on maximum loss to follow-up of 20%.

### Randomization

Randomization will be performed after the eligibility assessment and baseline assessment. The Department of Science and Technology of SUTCM will generate the randomization sequence using a random number generator (IBM, Chicago, IL, USA) and then placed in a sequenced, sealed, opaque envelope. When potential participants meet the inclusion criteria, the envelope can be opened and the subject will accept the corresponding measures. Then, eligible patients will be randomly divided into a guideline therapy group and a guideline therapy plus TCMR group, with an allocation proportion of 1:1.

### Blinding

Participants and therapists are unable to be blinded regarding group assignments due to the specific intervention, but the therapists will be masked to the evaluation of outcomes. To reduce the risk of bias, the outcome assessors, data managers, and statisticians will be unaware of the group assignments during the outcome evaluation and data analysis process. They will be required not to share study information. The blinding procedure will be in place until the data are locked.

### Data collecting and monitoring

The administrators at the Science and Technology Department of SUTCM will be responsible for monitoring data management as an independent third party. Paper case report forms (CRFs) will be designed to collect test data according to the trial protocol, which will be stored in a securely locked location. We will conduct a double independent data entry to promote data quality. Then, it will be locked and analyzed by an independent statistician under the supervision of the administrators. The test data will be recorded on the sub-website of China Clinical Trial Center (http://www.medresman.org.cn/login.aspx) electronic data management system. The electronic database will be closed after data entry is completed. The administrators report the data supervision to the steering committee every week, mainly including the reliability of the data and the accuracy of the entry.

### Statistical analysis

#### General analysis principles

Statistical analysis will be performed using IBM SPSS version 25.0 (IBM Corp., Armonk, NY, USA). A Kolmogorov-Smirnov test with Lilliefors correction will be used to analyze all quantitative variables to determine whether they follow a normal distribution. Nonnormally distributed data will be expressed as the median (upper and lower quartiles), and normally distributed data will be expressed as the mean ± the standard deviation (SD). Categorical variables will be presented as frequencies and percentages. The level of significance will be *α* < 0.05 with a two-tailed test, and 95% confidence intervals will be presented where appropriate.

#### Analysis of primary outcome

The primary outcomes will be evaluated at baseline, 6 days after the intervention, and upon discharge based on the intention-to-treat analysis (ITT). The primary endpoint of the trial is the mean change in mMRC Scales from baseline to the discharge day. Baseline covariates included for adjustment were sex, age category, history of cardiopulmonary disease, and body weight category. The whole outcomes scale was also measured at baseline. When the data have a normal distribution, two-way repeated-measures ANOVA will be used as the main analytic method, and a paired sample *t* test will be used between the two intervention groups; if the normal distribution is not met, the Wilcoxon test will be used as an alternative method. Prior to any analysis, any missing data pattern will be investigated and reasons for missing data obtained and summarized where possible. The percentage of missing data is 20%, and we plan to generate 5 imputed datasets, which is the minimum recommended number [28]. The sensitivity analyses were carried out to assess two assumptions about missing data, and data imputation scenarios can potentially influence the primary result [29]. To account for missing data, our primary analyses were conducted on an imputed data set where missing values were generated using multiple imputation by chained equations (MICE) [30].

#### Analysis of secondary outcomes

Analysis of secondary outcomes will be undertaken using a similar approach to that described for the primary analysis. We will estimate and test for a difference between treatment arms for each endpoint specified in the secondary outcomes listed above. Adverse events will be listed and analyzed using the chi-square test or Fisher’s exact test.

### Interim analysis

#### An interim analysis will not be conducted

##### Quality control

During the whole processing of the trial, quality control will be conducted under the surveillance of the steering committee. To ensure the consistency of methods, all the researchers will be trained with the trial methodology and monitoring technique before participating in the trial. Supervision activities will be divided into online remote monitoring and offline personal inspection every day. If necessary, researchers will be able to submit a request to modify the protocol, such as the primary outcome and sample size calculation. The steering committee and ethics committee will have the authority to allow modification of the plan. In addition, the information will be updated in the clinical registration center in time to ensure that all parties can understand the changes.

##### Safety evaluation

A safety evaluation will be conducted throughout the trial. Although TCMR programs are low risk, the participants are ill with severe pneumonia. When adverse events occur, the researchers will record them in CRFs in detail and analyze whether they are directly related to the rehabilitation program. Regardless of the cause, if the condition suddenly worsens during the trial and is accompanied by severe complications or serious adverse reactions, the trial will be terminated immediately, and prompt medical measures will be taken according to the subject’s condition. The specialist will be prepared to deal with some harm during the study at any time, with treatments including oxygen therapy support, venous access, and reasonable medication to maintain normal vital signs.

## Discussion

The city of Wuhan in China is the focus of global attention due to the outbreak of COVID-19 [31], which has an epidemiological link to the Huanan Seafood Wholesale Market. Although the overall mortality rate is relatively low compared to other coronavirus epidemics [32], the infection has worsened due to encounters from mass migration [33]. Immediate medical measures and rescues by the Chinese Government have been taken to control further deterioration of the epidemic; these include intensive surveillance, epidemiological surveillance, and symptomatic supportive treatment to reduce mortality. Though all the costs of COVID-19 treatment are covered by medical insurance in China, there are still no effective therapies or vaccines except for meticulous supportive care [34]. Therefore, it is necessary to find more measures to deal with this pneumonia. As a category of complementary and alternative medicine, acupressure therapy and Liu Zi Jue Qigong exercises are performed widely in TCM [35, 36]. Some researchers have confirmed that they can improve the clinical symptoms and physical and mental health of patients with lung disease [10, 37, 38]. As the health care system is overloaded, a TCMR program is highly operable and cost-effective [39]. It is a low-risk therapy that does not require large equipment and is not restricted by location or time [40]. Through TCM rehabilitation, the body’s immune system can offset the damage of foreign microorganisms through the dynamic regulation of innate immunity [41]. Previous studies have found that these points can significantly improve lung symptoms, such as cough, chest tightness, and sputum [42]. In addition, Liu Zi Jue Qigong breathing exercises can help restore the physiology of the lungs to diffuse and sink (inhale and exhale). This technique is a pursed-lip breathing and inspiratory muscle training method that can increase muscle strength and respiratory muscle endurance to relieve dyspnea. During the performance of the low-intensity exercise, patients are focused on themselves, which can relieve some anxiety and tension attributed to a negative situation [43]. According to the TCM macro theory, the TCMR program can harmonize qi and blood coordination and regulate the balance of yin and yang. However, more strong evidence is necessary to further prove the efficacy of TCMR programs for improving the symptoms and prognosis of patients with COVID-19. Therefore, this clinical trial was conducted to evaluate the efficacy of a TCMR program including acupressure and Liu Zi Jue Qigong exercises to improve pneumonia and quality of life of patients with COVID-19.

### Trial status

This trial is recruiting patients now. Participant recruitment started on 20 February 2020 and is expected to end on 30 June 2020. This trial was registered in the Chinese Clinical Trial Registry on 18 February 2020. The registration number is ChiCTR2000029994.

Protocol version 3, 8 July 2020.

## Supplementary information

**Additional file 1.** SPIRIT 2013 Checklist: Recommended items to address in a clinical trial protocol and related documents.

## Data Availability

The protocol manuscript does not contain any data, so we declare that data and materials are not applicable in this study. The datasets generated and analyzed during the study will be available in the Figshare repository.

## Abbreviations

COVID-19: Novel coronavirus pneumonia
ADL: Activities of daily living
CRF: Case report form
mMRC: Modified Medical Research Council
PHQ-9: Patient Health Questionnaire-9
SUTCM: Shanghai University of Traditional Chinese Medicine
RS: Respiratory symptoms
TCMR: Traditional Chinese medicine rehabilitation
